# Does Vaterite Otolith Deformation Affect Post-Release Survival and Predation Susceptibility of Hatchery-Reared Juvenile Atlantic Salmon?

**DOI:** 10.3389/fvets.2021.709850

**Published:** 2021-09-27

**Authors:** Aurélien Delaval, Martine Røysted Solås, Helge Skoglund, Anne Gro Vea Salvanes

**Affiliations:** ^1^Department of Biological Sciences, University of Bergen, Bergen, Norway; ^2^Faculty of Biosciences and Aquaculture, Nord University, Bodø, Norway; ^3^Laboratory of Freshwater Ecology and Inland Fisheries, NORCE Norwegian Research Centre, Bergen, Norway

**Keywords:** enrichment, fish stocking, otolith deformities, Salmo salar, vaterite

## Abstract

Sagittal otoliths are calcareous structures in the inner ear of fishes involved in hearing and balance. They are usually composed of aragonite; however, aragonite can be replaced by vaterite, a deformity which is more common in hatchery-reared than in wild fish. Vaterite growth may impair hearing and balance and affect important fitness-related behaviours such as predator avoidance. Captive rearing techniques that prevent hearing loss may have the potential to improve fish welfare and the success of restocking programmes. The aim of this study was to test the effect of structural tank enrichment on vaterite development in the otoliths of hatchery-reared juvenile Atlantic salmon *Salmo salar*, and to assess the effects of vaterite on immediate predation mortality and long-term survival after release into the wild. Fry were reared in a structurally enriched or in a conventional rearing environment and given otolith marks using alizarin during the egg stage to distinguish between the treatment groups. Otoliths were scrutinised for the presence and coverage of vaterite at 6, 13, and 16 weeks after start feeding, and the growth traits were measured for enriched and control fry when housed in tanks. In a subsequent field experiment, juveniles were released in the Rasdalen river (western Norway), and otoliths of enriched reared and control reared fry were scrutinised from samples collected immediately prior to release, from predator (trout *Salmo trutta*) stomachs 48 h after release and from recaptures from the river 2–3 months after release. Vaterite otoliths occurred as early as 6 weeks after start feeding in hatchery-reared *S. salar*. Vaterite occurrence and coverage increased with fish length. Enriched rearing had no direct effect on vaterite formation, but enriched reared fry grew slower than control fry. After release into the wild, fewer salmon fry with vaterite otoliths had been eaten by predators, and a higher proportion of fry with vaterite otoliths than those lacking vaterite were recaptured in the river 2–3 months after release. Contrary to expectations, this suggests that vaterite does not increase predation mortality nor reduce survival rates in the wild during the early life stages.

## Introduction

The inner ear of bony fishes contains calcareous structures (otoliths) that are part of the organs for hearing and balance. In teleost fishes, the largest pair of otoliths (the sagittae) are usually composed of a polymorph of calcium carbonate called aragonite. However, substitution of aragonite by vaterite, an alternative polymorph, has been documented in several species (1–7). Vaterite otoliths are larger, are deformed and have a lower density than aragonite otoliths (3, 6, 8). While relatively rare in wild fish, vaterite deposition is very common in hatchery fish and in aquaculture (2, 4, 6). Previous studies suggest that the presence of vaterite may impair hearing in salmonids (6, 8) and alter the escape kinematics (9) in salmonids as young as 6 months old.

The functional mechanisms underlying vaterite deposition and its consequences are largely unknown; hormonal (5), genetic, or biochemical (1) factors have been hypothesised as predictors, and there is growing evidence of the roles of different proteins in polymorph deposition at the molecular level (10). Vaterite prevalence during conventional hatchery rearing is reportedly associated with stress related to stocking density and handling practises (4), and with the typically faster growth rates mediated by diet, longer photoperiods that allow for continuous feeding, and temperature regimes (11). Consequently, vaterite is over 10 times more common in farmed than in wild fish, as demonstrated in a review spanning several species including a range of salmonids (6).

Hearing and balance are important sensory systems for fish's detection of auditory cues and for manoeuvring in a three-dimensional water body. Impaired hearing may generate biased soundscape-dependent swimming behaviour and challenge the welfare of captive fish. If the presence of vaterite is high in hatchery fish reared for release into natural habitats, impaired hearing may also bias the perception of predation risk and prey presence, sensory cues that are important for survival and growth (6, 9, 11).

Are there ways to reduce otolith deformities in hatcheries? Any captive rearing technique that prevents hearing loss in fish may have the potential to improve fish welfare. Growing research efforts in the last decades have shown that physical enrichment can improve fish welfare. Structural tank enrichment has been used as a method of improving behavioural phenotypes in juvenile fish reared for restocking programmes aiming to improve their chances of survival after being released into the wild (12–20). This means that captive fish are provided with more stimuli than in conventional tanks by increasing their structural complexity (e.g., provision of shelters). Field studies have shown mixed results regarding the effect of tank enrichment on the post-release survival of salmonids (21–25), suggesting that the relationship may be complex. Our research group has previously conducted field experiments to test whether structurally enriched rearing of Atlantic salmon *Salmo salar* reduced immediate (within 2 days after release) predation mortality by piscine predators (brown trout *Salmo trutta*), and improved long-term (2–3 months after release) post-release survival, and found that enrichment did not consistently reduce predation mortality or improve long-term fry survival (25). When scrutinising the otoliths during the study, which was required to distinguish between the treatment groups as determined by the number of alizarin marks given during the egg stage, we observed variations in the occurrence and extent of vaterite coverage among individual fish.

In the present paper, we have scrutinised otoliths from the same field experiment as of Solås et al. (25), and from enriched and control reared fish sampled in the rearing tanks on three occasions (July, August, September). We tested the effect of structural enrichment on vaterite presence and coverage using data from rearing tanks, and the effect of vaterite on survival using predation and release–recapture field experiments. In doing so, we could evaluate whether structural tank enrichment may represent a practical means to mitigate otolith deformities in juvenile hatchery fish, and whether the documented negative effects of vaterite on salmonid perception would translate to increased short-term predation mortality and reduced survival 2–3 months after release into the wild. Should the rearing of captive fish in structurally enriched environments reduce the incidence of otolith deformities and confer improved survival in the wild, the results could have implications for restocking programs and the aquaculture industry.

## Materials and Methods

### Ethical Statement

All research was approved by the Norwegian Food Safety Authority in compliance with “The Regulation on the Use of Animals in Research” with FOTS id 7931.

### Rearing and Experiments

We used juvenile Atlantic salmon *Salmo salar* reared at the Voss hatchery using eggs originating from the Vosso River population housed at the Haukvik live gene bank. Individuals were group-marked in the otoliths at the eyed egg stage using Alizarin Red-S, as described in Solås et al. (25), allowing us to differentiate between fry that would later be reared in an enriched rearing tank or in a conventional control rearing tank. Individuals of the enriched group were marked with two alizarin rings in their otoliths, while those in the control group were marked with one.

**Figure 1:**
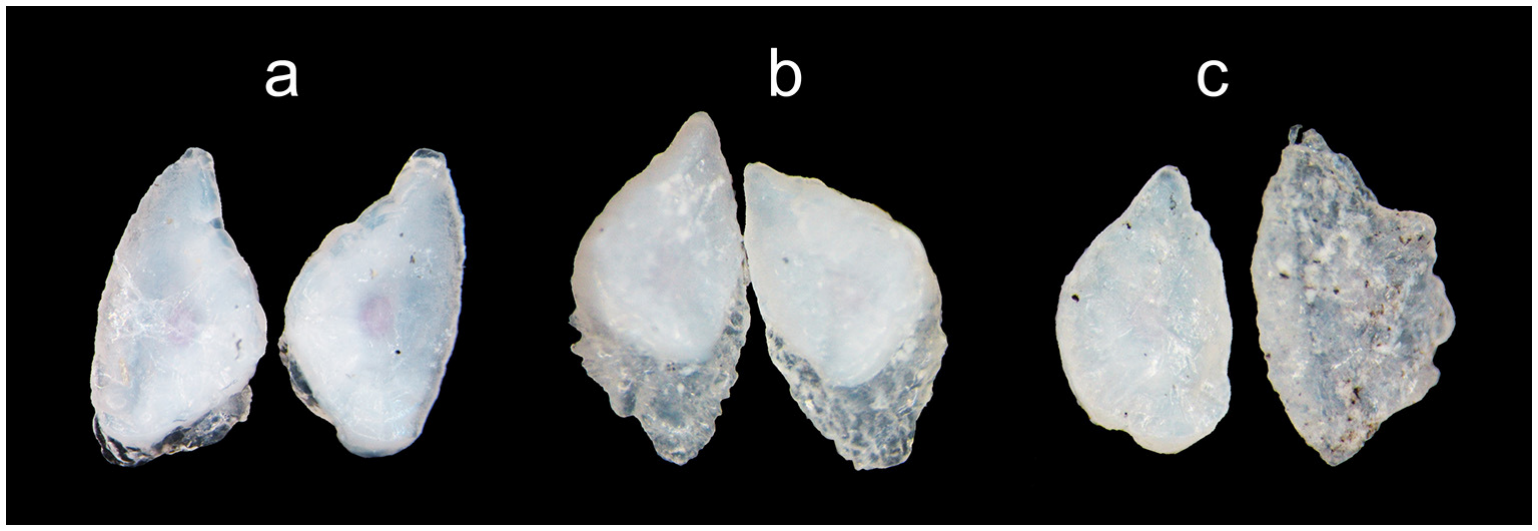
Pictures of vaterite otoliths from fry consumed by trout predators 48 h after release into the Rasdalen river in 2016. **(a)** Vaterite deposition just started; **(b)** medium vaterite cover; and **(c)** nearly total vaterite cover for the right otolith.

Hatching occurred around April 20^th^ in each of the years 2014, 2016, and 2017. The fry were transferred to two large tanks (2 × 2 m) after yolk sac absorption, where they received natural river water from the Vosso river. The first feeding was 1–2 weeks after the fish had been transferred to the rearing tanks. The fish were fed under continuous light from above, with commercial pellets (Nutra XP, Skretting, www.skretting.com) dispensed five times per hour at the water surface by an automatic feeder and increasing pellet size with fish size. Structural enrichment was introduced to one of the tanks (hereafter referred to as enriched) at the onset of feeding (hereafter called start feeding), while the fish in the other tank continued being reared in conventional hatchery environments (no additional structures, hereafter referred to as control). The enrichment consisted of four plastic tube constructions and one green box to provide shelter, both with nylon ropes and plastic shreds attached, to simulate river flora [Figures 1A,B of (25)]. The structures were cleaned approximately every other week during rearing in June and every week during rearing in July and August. Samples of fry from the tanks and at recapture were euthanised by an overdose of buffered MS222 (0.5 g l^−1^) and frozen at −20°C until they were processed.

#### Pre-release Tank Fish

Pre-release juveniles from 2014, 2016, and 2017 were used to study vaterite presence and vaterite coverage in the early life stages of fish from tank rearing. In 2014, the rearing tanks were randomly sampled three times: 6, 13, and 16 weeks after start feeding (Table 1). This allowed us to study the effect of time after start feeding and tank treatment on the probability of vaterite present and the vaterite coverage in the sagittal otoliths. We also used the data on juveniles from 2014 to test if enrichment had an effect on growth traits.

**Table 1:** The number of Atlantic salmon fry lacking vaterite (vaterite absent) in otoliths and the number of fry having vaterite (vaterite present) in at least one otolith for the tank data from 2014 at 6, 13, and 16 weeks after first feeding, pre-release (2016 and 2017), in fry eaten by trout predators 2 days after release into Rasdalen river (2016 and 2017) and at recapture from electrofishing 2–3 months after release in Rasdalen (2016 and 2017).

	**Vaterite absent**		**Vaterite present**	
	***N***	**Mean SL (cm)**	***N***	**Mean SL (cm)**
**2014: Tank data**				
**Date: 6 weeks after first feeding**				
Control	38	3.2 (0.4)	11 (0)	3.3 (0.3)
Enriched	41	3.4 (0.3)	9 (0)	3.2 (0.3)
**Date: 13 weeks after first feeding**				
Control	36	5.6 (1.2)	11 (4)	6.0 (1.2)
Enriched	40	5.5 (1.0)	15 (3)	5.9 (0.9)
**Date: 16 weeks after first feeding**				
Control	32	7.5 (1.7)	17 (1)	8.4 (1.4)
Enriched	41	7.4 (1.2)	9 (2)	7.0 (1.4)
**2016: Pre-release**				
Control	44	5.0 (0.5)	6 (1)	5.2 (0.6)
Enriched	41	4.7 (0.8)	4 (0)	6.0 (0.5)
**2016: Predator stomachs**			
Control	101	4.6 (0.5)	9 (0)	5.0 (0.4)
Enriched	69	4.6 (0.6)	6 (1)	4.9 (0.8)
**2016: Recapture**				
Control	9	5.3 (0.3)	27 (19)	5.5 (0.4)
Enriched	17	4.9 (0.6)	19 (13)	5.6 (0.5)
**2017: Pre-release**				
Control	25	5.7 (0.7)	21 (3)	5.9 (0.6)
Enriched	36	5.5 (0.8)	12 (3)	5.6 (0.9)
**2017: Predator stomachs**				
Control	11	5.3 (0.7)	5 (1)	5.6 (0.6)
Enriched	15	5.3 (0.5)	5 (0)	5.3 (0.7)
**2017: Recapture**				
Control	39	6.2 (0.5)	30 (4)	6.3 (0.5)
Enriched	27	6.0 (0.5)	12 (1)	6.0 (0.8)

*The numbers in parentheses of mean SL are standard deviations of SL, and the number in parentheses of N refer to the number of the N salmon fry having two vaterite otoliths*.

In 2016 and 2017, the tanks were only sampled the day of release of fry into the river, which was 13 weeks after start feeding. We used these fry, together with the ones sampled 13 weeks after start feeding in 2014, to test the effect of fish size, tank treatment, and year on the probability of vaterite presence and the vaterite coverage in sagittal otoliths of fry 13 weeks after start feeding.

#### Predation Field Experiment

A predation field experiment was carried out in the Rasdalen river in western Norway in 2016 and 2017. In each of the years, a total of 3,600 individuals (1,800 from each treatment) were transported to Rasdalen and released into the river mid-August. The fish were aged 17 and 16 weeks when released in 2016 and 2017, respectively. At the day of release, we collected a random subsample of ca. 100 individuals from both the control and enriched rearing tanks to obtain pre-release fish size and otolith compositions (these were the same individuals as described under Pre-release tank fish, for 2016 and 2017).

The release area in Rasdalen was located upstream of a migration barrier and consequently did not have any other Atlantic salmon individuals apart from potential older year-classes from egg boxes set out in 2013 and 2014. No wild-born salmon occur in this area, but it does have a natural population of resident brown trout (*Salmo trutta*). The river stretch was ~100 m long with an area of ~1,230 m^2^ and consisted of riffles, runs, and pools. The substrate was mainly composed of large stones and small boulders. Larger resident brown trout (standard length >10 cm) were considered as potential predators of the fry and were sampled 48 h after the release of fry. The trout were sampled by two people using point electrofishing with battery-powered backpack generators with a pulsed current of 1,400 V. It took 30–60 min to cover the entire release stretch and a few additional metres downstream. The stunned predators were collected with hand nets and transferred ashore in containers of river water for further examination. To collect the salmon fry that had been consumed by the brown trout predators, the sampled trout were either anaesthetised with MS-222 to enable evacuation of stomach contents in the field or euthanised with an overdose of MS-222 for later examination of stomach contents in the lab (see (25) for details).

Juvenile Atlantic salmon from the stomach contents of anaesthetised trout were collected using the gastric lavage technique (26). We inserted a 60-ml syringe fitted with a thin aquarium tube into the mouth of the trout, to the distal parts of the stomach, and flushed out stomach contents with water onto a sieve. Stomach contents were then put in a cooler to slow the decomposition process and later frozen. The brown trout predators recovered from anaesthesia in a 30-l tank containing river water before being released back into the river.

#### Release-Recapture Field Experiment

The fry released into the Rasdalen river in August of 2016 and 2017 were recaptured 2–3 months later using the same electrofishing technique as described above. Fish were also released in 2014, but recaptures from that year had to be excluded due to extreme weather conditions in the autumn. The Vosso river system had a 200-year-flood and the recaptures late in the autumn were not considered representative, and only data from before release in 2014 are included in the present paper. The whole release stretch and additional 50 m downstream were sampled (to sample fry that had dispersed downstream) with the aim of recapturing ~100 fry released 2–3 months earlier.

### Measurements and Otolith Examination

All fish were measured (standard length, to the nearest 0.1 cm) and weighed (to the nearest 0.01 g) prior to extracting their sagittal otoliths. Otoliths were cleaned of adhering tissue and air-dried, and their distal surface was photographed under a Leica M125 stereo microscope mounted with a Nikon Digital Sight DS-Fi2 camera at ×40 or ×50 magnification depending on otolith size such that the length of the otolith filled >30% of the field of view. Of the fry from the rearing tanks in 2014, 2016, and 2017, ~50 individuals from each of the enriched and control treatments were examined for the presence and proportion cover of vaterite (Table 1). The presence of vaterite on the otoliths was noted before or after they were polished to categorise fry into treatment groups (enriched and control, see section about treatment groups below) and using a microscope.

Our first notice of vaterite was a serendipitous discovery, and it occurred after a large proportion of the otoliths from 2017 had been polished. The otoliths had to be polished for the alizarin marks to be visible, which was necessary to identify which fry had been reared in enriched and control tanks. Some otoliths do have a thin clear outer region, which may or may not indicate the onset of vaterite development. Two observers scored the otoliths for presence/absence of vaterite, and where they disagreed, a conservative approach was taken and vaterite was regarded as absent. For all otoliths, vaterite was not regarded as present unless crystallisation was clearly visible (Figure 1). Presence/absence scoring was done on all otoliths except for a handful that had to be excluded from the analysis as they were difficult to interpret (e.g., shattered while handling).

Coverage of the two-dimensional distal surface area of an otolith composed of vaterite (hereafter denoted vaterite coverage) was measured on the photos, except for fry from predator stomachs from 2017, to a precision of 1% (i.e., 0.01) using ImageJ (ver. 1.46r). For fry from predator stomachs from 2016, photos of otoliths were taken both before and after polishing the otoliths. These were used to evaluate whether we could measure the coverage of vaterite in polished otoliths from 2017. We decided to use a conservative approach for these otoliths by categorising them binomially by considering the otolith to either have vaterite coverage ≤5% or have vaterite coverage >5%. This approach was also applied to the pre-release otoliths for 2016 and 2017 so that we could compare the probability of vaterite coverage >5% of fry consumed by predators with that of fry pre-release for both 2016 and 2017.

### Identifying Treatment Groups From Otoliths

Control and enriched rearing background of fry from predator stomachs and recaptures in the river was determined by examining otoliths for fluorescent alizarin rings. The otoliths were fixed on individual slides using temporary mounting wax (CrystalBond; www.aremco.com, or Quick-Stick; www.innovatekmed.com), and then otoliths were polished with grinding paper until the daily increments of otoliths became visible (27). Next, we identified the number of fluorescent rings using an epifluorescent microscope (Zeiss Axioskop 2 Plus, www.zeiss.com) and UV light. Control fish had one fluorescent ring whereas enriched fish had two.

### Statistical Analysis

All statistical analyses were performed in R (ver. 3.6.2, www.r-project.org), and plots were produced using the additional package ggplot2 (28). The data were organised such that each fry was regarded as having vaterite present if at least one otolith contained vaterite. In cases where both otoliths had vaterite (Table 1), the otolith with the highest vaterite coverage was included in the statistical analysis. Since the majority of samples had only one affected otolith, we decided not to take the mean vaterite coverage of both otoliths so as not to generate a bias for the few samples with two affected otoliths. To ensure no effect of this on the results, we performed exploratory analyses taking the mean vaterite coverage from both otoliths, which had a minimal effect on the results (data not shown).

For the pre-release tank fish sampled at the same age among years (13 weeks after start feeding), we used generalised linear models (GLMs, logit link) to test for the effects of standard length (cm), treatment (control or enriched rearing), and year (2014, 2016, or 2017) on the probability of vaterite presence and vaterite coverage. Standard length was specified as continuous and treatment and year as categorical effects.

For the pre-release tank fish from 2014 sampled at different weeks after start feeding (6, 13, and 16 weeks), we used GLMs (logit link) to test for the effects of time (weeks) after start feeding and treatment on the probability of vaterite presence and vaterite coverage. Time after start feeding was specified as continuous. A linear model was applied to the data on standard length using log-transformed data to test if enrichment had effect on growth.

For the predation field experiment, we pooled the two treatments (enriched and control) and used GLMs (logit link) to test the effects of standard length and sample group (pre-release or predator stomach) on the probability of vaterite coverage >5% vs. vaterite ≤5%. Standard length was specified as a continuous variable and sample group as a categorical effect. We assumed quasibinomial distributions to account for over dispersion in the data for all the GLMs.

In addition, a Pearson's chi-test was used to evaluate whether the proportion of individuals with vaterite otoliths differed between fry sampled from the tanks the day of release into the river and those sampled from predator stomachs 48 h after release. The chi-test was also used to evaluate whether vaterite occurrence differed between fry sampled before release and those recaptured from the river 2–3 months after release. Since the proportion of fry with vaterite decreased from pre-release to predator stomachs and increased from pre-release to recapture 2–3 months later both in 2016 and in 2017 (Table 1; **Figure 4**), we pooled the data from these 2 years for both tests.

## Results

Vaterite was detected in the sagittal otoliths of fry after hatchery rearing in all 3 years. Of a total of 890 fish that were scrutinised, vaterite was present in at least one of the otoliths in 228 fish (25.6%), whereas 56 fish (6.3%) had vaterite in both otoliths. However, the extent of vaterite occurrence varied among years, sampling time, and treatment (Table 1), which could be a result of random sampling effects.

**Figure 2:**
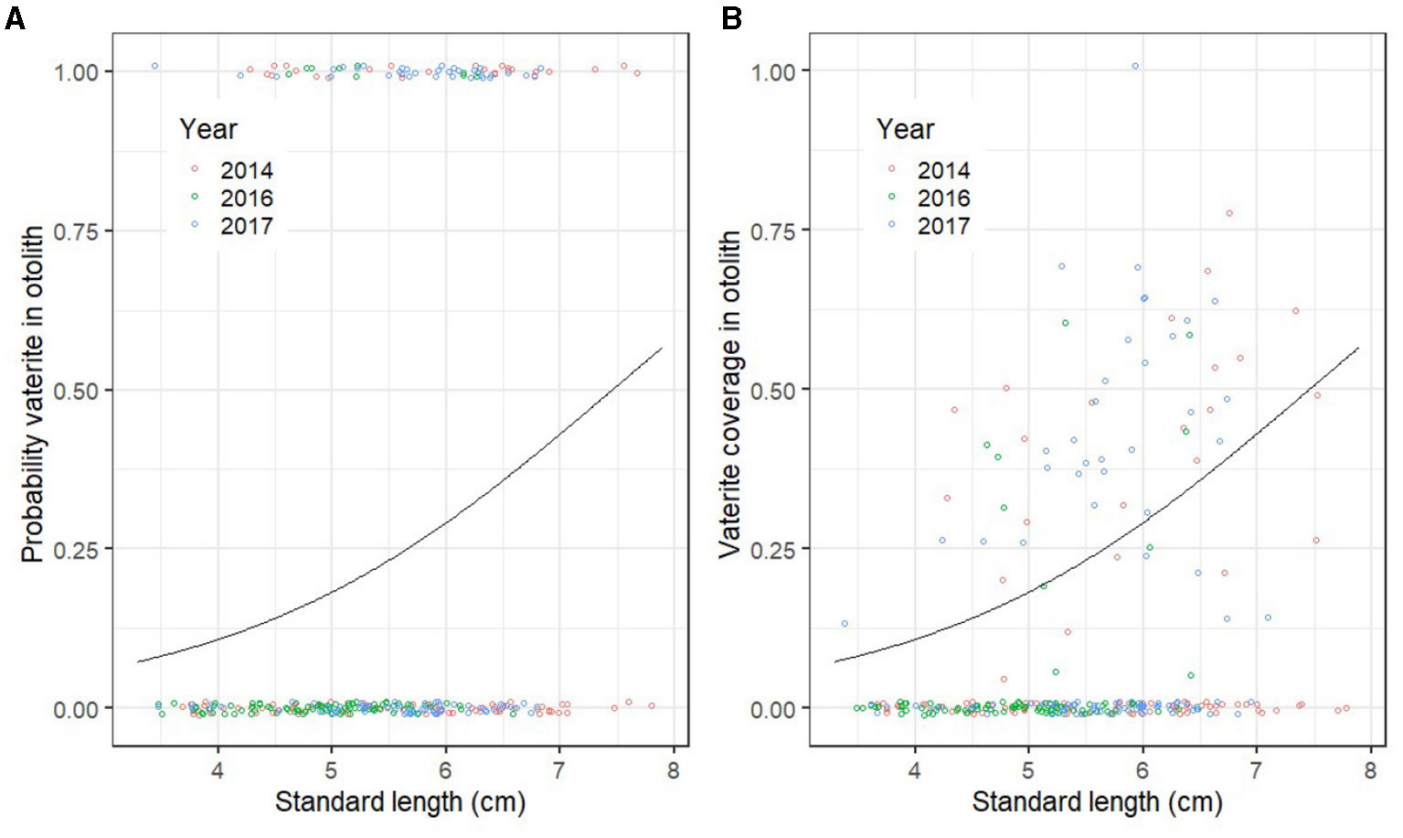
**(A)** Probability of vaterite present in at least one otolith by standard length (cm) in Atlantic salmon fry ca. 13 weeks after first feeding in 2014, 2016, and 2017 (the three treatments have been pooled). **(B)** Vaterite coverage of the otolith with most vaterite as a function of standard length (cm). The points show the raw data, which are jittered to allow overlapping points to show. The lines represent model predictions generated by retransforming the parameters from logistic regression.

When comparing pre-release fish that were sampled at the same age among years (13 weeks after start feeding), the probability of vaterite being present in at least one otolith increased with fry length (GLM; deviance = 15.86; *p* << 0.01; Figure 2A), but did not differ between 2014, 2016, and 2017 (GLM; deviance = 0.55, *p* = 0.47). There was no main effect of treatment on the probability of vaterite being present (GLM; deviance = 1.27, *p* = 0.27), but a marginally non-significant interaction effect of year and treatment (GLM; Deviance = 3.12; *p* = 0.08). The vaterite coverage of the otoliths also increased with length (GLM; deviance = 7.51; *p* << 0.01; Figure 2B) and did not differ between years (GLM; deviance = 0.43, *p* = 0.32; Figure 2B). There was a marginally non-significant effect of treatment on vaterite coverage (GLM; deviance = 1.61, *p* = 0.054).

**Figure 3:**
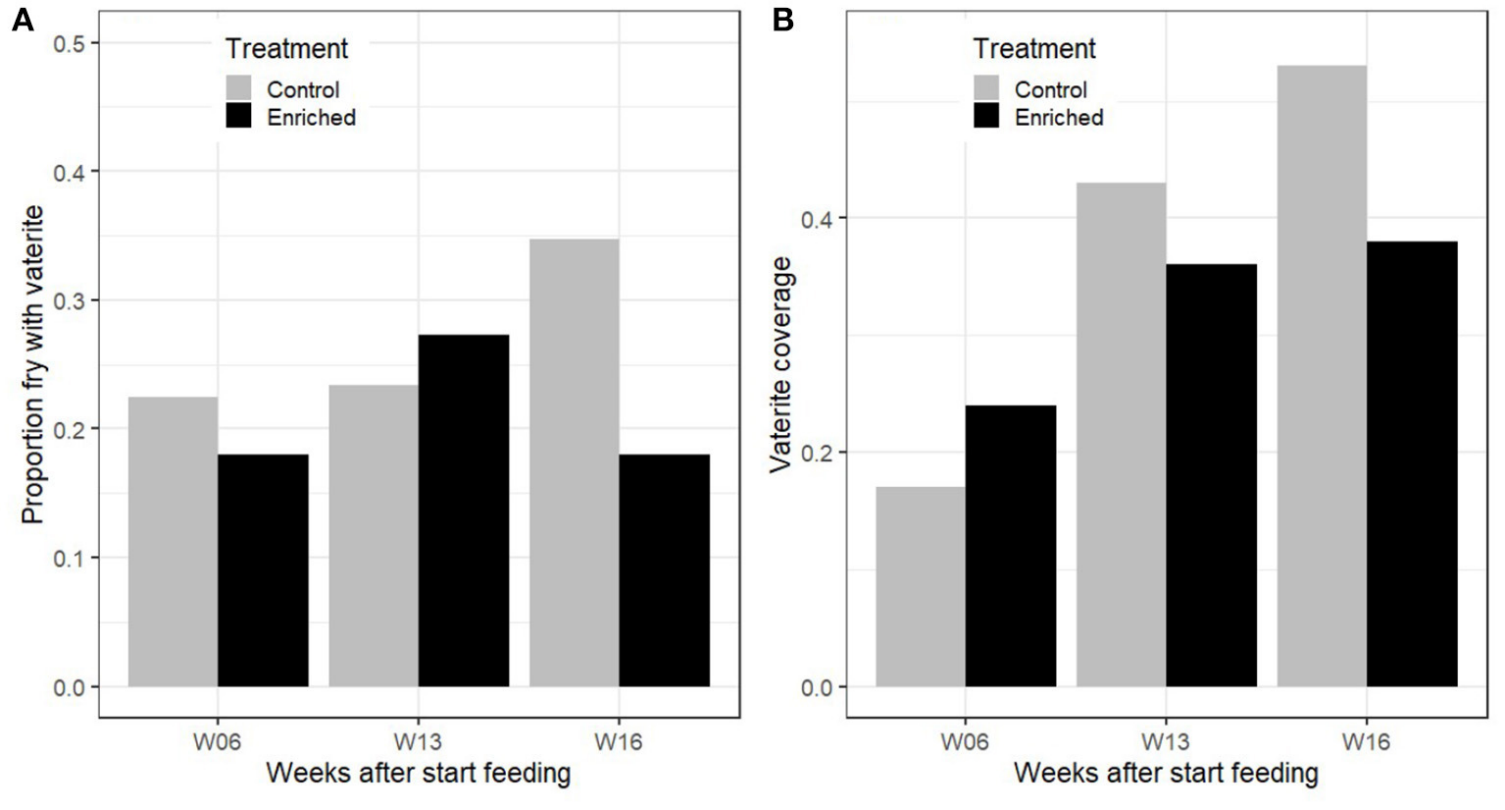
2014 Tank data. **(A)** Proportion of control and enriched reared salmon fry that had at least one vaterite otolith at 6, 13, and 16 weeks after first feeding. **(B)** Mean proportion vaterite coverage of otoliths, excluding otoliths without vaterite.

For the 2014 tank fish sampled at different times after start feeding, vaterite was detected in both control and enriched reared fry and as early as 6 weeks after start feeding. Overall, 72 out of 300 fish sampled from the tanks in 2014 had detectable vaterite in at least one sagittal otolith. The maximum proportion of an otolith covered by vaterite in control fry was 0.37 at 6 weeks after start feeding and increased to 0.84 and 0.83 at 13 and 16 weeks, respectively. For enriched fry, the maximum proportion cover was 0.39 at 6 weeks after start feeding and increased to 0.69 and 0.76 at 13 and 16 weeks, respectively. There was no significant effect of treatment on the probability of vaterite present in at least one of the otoliths from the 2014 tank data (GLM; deviance = 1.29, *p* = 0.26), and there was no change in the probability of vaterite presence from 6 to 16 weeks after start of feeding (GLM; deviance = 1.18, *p* = 0.28; Figure 3A). However, the vaterite coverage of affected otoliths increased with time after start feeding (GLM; deviance = 10.64, *p* << 0.001; Figure 3B). There was no main effect of treatment on otolith coverage (GLM; deviance = 0.45, *p* = 0.22).

The standard length of fry from both treatments increased significantly over time after start feeding [LM; *F*_(1, 296)_ = 33.58, *p* << 0.001], but the enriched reared fry had a slower growth than control fry between 6 and 16 weeks after first feeding (LM; interaction of treatment^*^weeks since first feeding; *t* = −2.20, *p* = 0.028.

**Figure 4:**
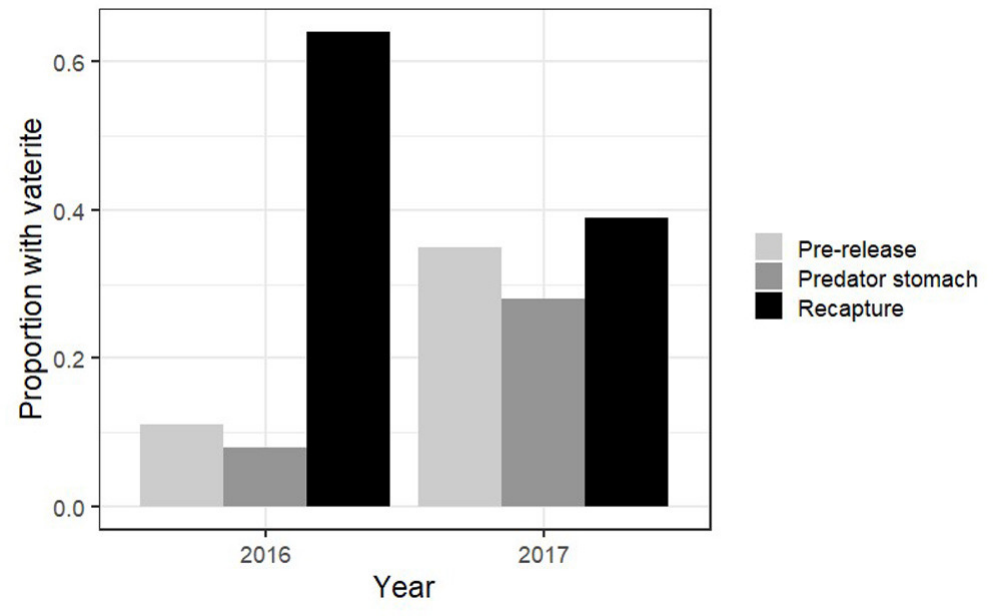
Predation experiment and release–recapture experiment. Proportion of Atlantic salmon juveniles that had at least one vaterite otolith present pre-release, predator stomachs 48 h after release and 2–3 months after release in 2016 and 2017.

**Figure 5:**
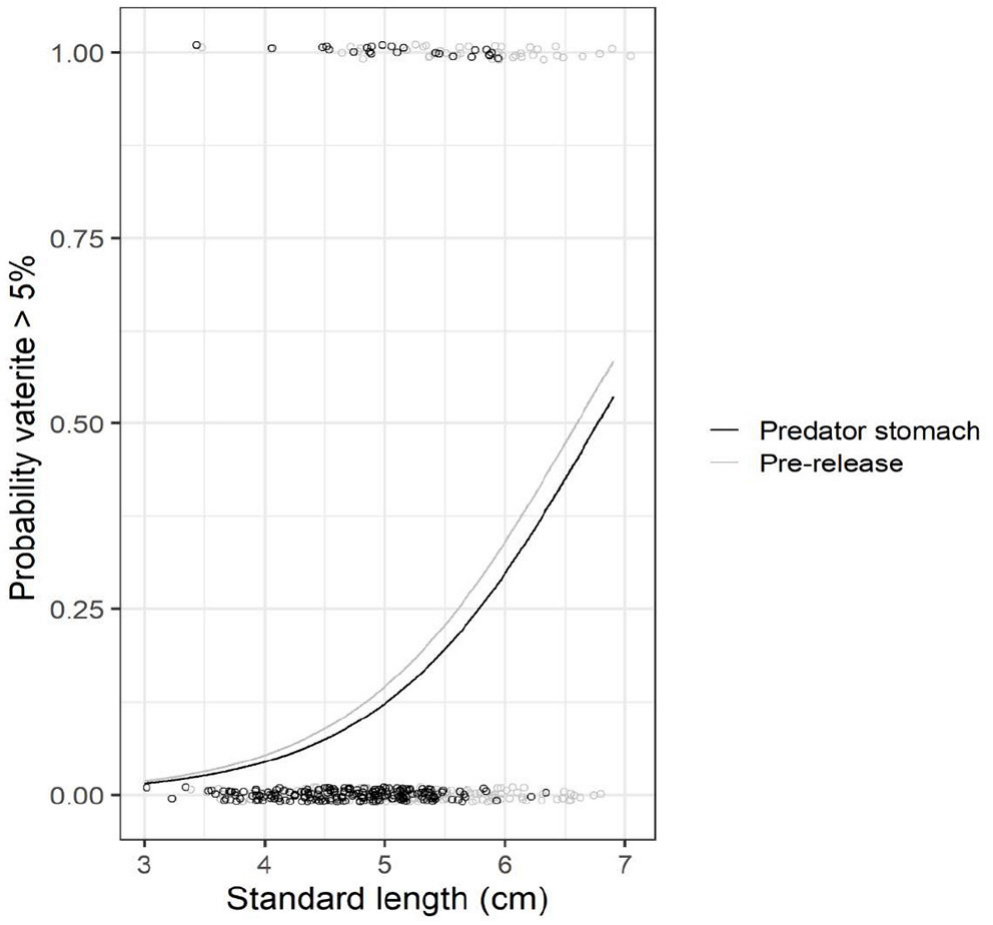
Predation experiment. Prevalence of >5% vaterite in at least one otolith with increasing size of reared Atlantic salmon fry. Data from 2016 and 2017 are pooled. Black circles refer to observed data for fry from the predator stomachs, and grey circles to fry pre-release. The data points are jittered to allow overlapping points showing. The lines represent model predictions that were generated by retransforming the parameters from logistic regression.

From the predation field experiment, the pooled data from 2016 and 2017 show that the proportion of fry with vaterite present was lower in predator stomachs (11% of fry) than pre-release 48 h earlier (23% of fry, chi-test: ${\text{X}}_{1}^{2}\;=\;9.29;p=0.002$; Figure 4). The probability that at least one otolith of a fry had >5% vaterite coverage increased with fry size (GLM; deviance = 28.88; *p* << 0.001; Figure 5), and the probability was lower for fry in predator stomachs than pre-release 48 h earlier (GLM; deviance = 9.43, *p* = 0.003; Figure 5).

The pooled data from the release–recapture field experiment in 2016 and 2017 show that survival was higher in released fry with vaterite otoliths 2–3 months after release than in fry without vaterite (chi-test; $X_{1}^{2}=27.5,\;p\ll 0.001$; Figure 4).

## Discussion

The increased occurrence of otolith aberrations due to vaterite formation in captive-reared fish has recently received increased attention as its impact on hearing and balance may compromise fish welfare in aquaculture (6) and reduce the performance of hatchery fish released into the wild (29). In the present study, we demonstrate that vaterite formation may occur very early in the juvenile life stages of Atlantic salmon, and within the first 6 weeks after start feeding in hatchery tanks. We found no evidence that vaterite formation was directly affected by the structural enrichment of the rearing environment. However, vaterite was strongly associated with larger body sizes, suggesting that it may be linked to faster growth rates. After release into the wild, salmon fry with vaterite otoliths were not overrepresented in stomach samples of predatory brown trout but were rather underrepresented. Furthermore, salmon fry recaptured 2–3 months after release into the river had a higher occurrence of vaterite compared to samples at the time of release. Therefore, neither does vaterite necessarily result in increased susceptibility to predation nor does it necessarily affect mortality in the wild.

Vaterite replacement in sagittal otoliths occurs sporadically in the wild, but is typically 3–10 times more prevalent in captive-reared fish (4, 6). The strong relationship between captive rearing and increased vaterite occurrence has been attributed to stress caused by intense growth and rearing conditions in hatchery tanks (2, 4, 6). However, the underlying mechanism behind vaterite replacement is still unknown. Reimer et al. (11) found vaterite to be strongly related to rapid growth rates in Atlantic salmon juveniles and suggested that abnormally fast growth disrupts normal aragonite deposition and triggers replacement by vaterite in otoliths. This is in accordance with the results of the present study, where the extent of vaterite was strongly related to body size. However, Austad et al. (29) found no relationship between vaterite and body size in Atlantic salmon smolts but suggested instead that vaterite may have been influenced by crowding in tanks. Bowen et al. (2), on the other hand, found that the prevalence of fish with vaterite increased markedly after routine intervention including dip netting and weighing the fish, suggesting that handling stress may be the main cause. This suggests that vaterite may also be influenced by various physical and social conditions in the rearing tanks. We found no evidence of any direct effect of environmental enrichment, in the form of adding physical structures in the rearing tanks, on vaterite formation. However, enrichment appeared to have an indirect effect on vaterite; fish grew slower in the structurally enriched tanks than in the plain tanks (control treatment), corresponding to a somewhat lower proportion of vaterite in enriched tanks. The lack of growth effect on vaterite in the study of Austad et al. (29) may be due to the fact that size was measured at a later life stage (i.e., at the smolt stage vs. early juvenile stage as in the present study). Growth of juvenile Atlantic salmon may vary across the season, and individuals that experience slow growth initially may also show compensatory growth later in life (30). Any potential growth effect resulting in vaterite deposition during earlier life stages, when it would most likely have initiated, may therefore have been concealed by growth compensation during later juvenile stages. While the role of environmental factors in determining vaterite formation during tank rearing remains to be resolved, the present study strengthens the hypothesis of fast growth being a key factor in causing vaterite otolith deformation.

Most studies indicate that vaterite replacement predominantly begins during the first year of life (4). However, little is known about the precise age at which vaterite replacement is initiated and whether certain life stages are particularly sensitive to vaterite development. In the present study, vaterite was present in 20% of the fish already 6 weeks after the onset of feeding in the tanks during the 2014 tank data. There was little change in the occurrence of vaterite after 13 and 16 weeks of feeding in the 2014 tank data, indicating that vaterite growth was mostly initiated during the early stages of tank rearing. Similarly, Bowen et al. (2) found that the majority of vaterite development appeared to occur within the first 5 months of life in lake trout *Salvelinus namaycush*, and indications of early vaterite development were identified during the first weeks after the onset of feeding in rainbow trout *Oncorhynchus mykiss* (5). The initial weeks of the juvenile stage are often critical in fish; during this time, individuals often prioritise rapid growth and development to outgrow the prey size range of gape-limited predators (31). Although more research is needed to resolve when vaterite forms in fish, our study suggests that the first weeks of juvenile rearing may be particularly sensitive for vaterite development.

Otoliths are a key component in the sensory system of fish, contributing to hearing, balance, the detection of gravity, and linear acceleration (32). Hence, otoliths are crucial for the ability of fish to manoeuvre in their surroundings and respond to immediate threats. Vaterite replacement in otoliths has been shown to result in a significant loss of hearing functionality (6, 8) and to affect escape response (9) and is therefore likely to reduce the ability of young fish to detect and escape from predators. Surprisingly, we did not find fry with vaterite otoliths to be overrepresented in the stomach contents of natural predators sampled 48 h after release. On the contrary, they were underrepresented when compared with fry sampled before release. Furthermore, the prevalence of fish with vaterite was significantly higher in samples of fry recaptured after 2–3 months in the wild, suggesting that the survival rate of fry with vaterite was higher and not lower than fry with normal otoliths. The latter result is likely to be due to size selective mortality; fry with vaterite otoliths were significantly larger, whereas predation was found to be selective toward smaller fry (see also (25)). Thus, despite high predation mortality and a high scope for selection, body size appeared to have a stronger effect on survival than the presence of vaterite in the sagittal otoliths.

The apparent absence of an effect of vaterite on predation susceptibility and mortality in the wild suggests that the loss of hearing may be of minor ecological significance early in life, or that the fish can compensate for reduced otolith function. For example, fish may partially compensate for reduced hearing by using the lateral line system for stimuli that are in close proximity (9). Furthermore, hearing may be more important under certain environmental conditions or during different life stages. Juvenile salmonids largely rely on vision to search for food and to detect predators (33), but auditory cues and hearing may become more important when visual conditions are suboptimal. Hearing and manoeuvrability may also be more important during pelagic phases, for example during smolt migration in fjords and post smolt feeding in open oceans. Austad et al. (29) found that the proportion of vaterite was lower in returning Atlantic salmon adults than in released smolts, suggesting that vaterite may have a negative effect on marine survival. On the other hand, Sweeting et al. (4) found the prevalence of vaterite to be higher in returning coho salmon *Oncorhynchus kisutch* than in released smolts, suggesting that the effect on marine survival may not be ubiquitous, at least across species. Nor was vaterite found to affect homing ability in chum salmon *Oncorhynchus keta* (34). The evidence is therefore mixed, and more data are needed to elucidate the effect of otolith deformities on the performance of fish across different environments, species, and life stages in the wild.

The underlying causes of vaterite formation in the sagittal otoliths of fishes, and the consequences in terms of fish welfare and fitness, remain unclear. It is evident that the prevalence of vaterite otoliths is higher in captive-reared fishes and is associated with conditions that facilitate rapid growth. We have also demonstrated that deposition is likely to begin within the first few weeks after start feeding or earlier. Therefore, experimental studies manipulating the rearing environment during these early life stages may help to identify methods of reducing otolith deformities in hatchery-reared salmon. Although the consequences of vaterite otoliths for fish hearing and behaviour have been documented, the results from the present study suggest that vaterite does not necessarily translate to lower fitness for juveniles released into the wild. Recapture experiments spanning the lifespan of salmonids from hatchery to maturity may help to elucidate the long-term effects of otolith deformities on long-term fitness after release.

## Data Availability Statement

The raw data supporting the conclusions of this article will be made available by the authors, without undue reservation.

## Ethics Statement

The animal study was reviewed and approved by Norwegian Food safety Authority in compliance with the regulation on the Use of Animals in Research with FOTS ID 7931.

## Author Contributions

AS, AD, and MS designed the study. AS raised the funding. AD and MS did the lab work and examined fish otoliths under the supervision of HS. AD and AS analysed the data. AS, AD, and HS wrote the first version of the manuscript. All authors have commented on and discussed the results, and all have contributed with comments and the writing of the final version.

## Funding

We thank the Nansen Foundation, University of Bergen, and the Olav Thon Foundation for funding.

## Conflict of Interest

The authors declare that the research was conducted in the absence of any commercial or financial relationships that could be construed as a potential conflict of interest.

## Publisher's Note

All claims expressed in this article are solely those of the authors and do not necessarily represent those of their affiliated organizations, or those of the publisher, the editors and the reviewers. Any product that may be evaluated in this article, or claim that may be made by its manufacturer, is not guaranteed or endorsed by the publisher.
